# Genetic Susceptibility to ANCA-Associated Vasculitis: State of the Art

**DOI:** 10.3389/fimmu.2014.00577

**Published:** 2014-11-17

**Authors:** Francesco Bonatti, Michele Reina, Tauro Maria Neri, Davide Martorana

**Affiliations:** ^1^Unit of Medical Genetics, Laboratory of Molecular Genetics, Diagnostic Department, University Hospital of Parma, Parma, Italy

**Keywords:** ANCA-associated vasculitis, granulomatosis with poliangiitis, microscopic poliangiitis, eosinophilic granulomatosis with poliangiitis, pharmacogenetics, rituximab, genome-wide association studies

## Abstract

ANCA-associated vasculitis (AAV) is a group of disorders that is caused by inflammation affecting small blood vessels. Both arteries and veins are affected. AAV includes microscopic polyangiitis (MPA), granulomatosis with polyangiitis (GPA) renamed from Wegener’s granulomatosis, and eosinophilic granulomatosis with polyangiitis (EGPA), renamed from Churg–Strauss syndrome. AAV is primarily due to leukocyte migration and resultant damage. Despite decades of research, the mechanisms behind AAV disease etiology are still not fully understood, although it is clear that genetic and environmental factors are involved. To improve the understanding of the disease, the genetic component has been extensively studied by candidate association studies and two genome-wide association studies. The majority of the identified genetic AAV risk factors are common variants. These have uncovered information that still needs further investigation to clarify its importance. In this review, we summarize and discuss the results of the genetic studies in AAV. We also present the novel approaches to identifying the causal variants in complex susceptibility loci and disease mechanisms. Finally, we discuss the limitations of current methods and the challenges that we still have to face in order to incorporate genomic and epigenomic data into clinical practice.

## Introduction

Vasculitis is a group of disorders that is caused by inflammation affecting blood vessels; it is primarily due to leukocyte migration and resultant damage (1). It is a multi-system inflammatory-autoimmune disease that affects both arteries and veins with consequent tissue damage, especially to the respiratory tract and kidneys; it causes early mortality, organ failure including end stage renal disease and chronic morbidity. Children and adults, males and females, and individuals of any ethnic background can be affected.

According to the size of the vessel affected, vasculitis can be classified into large, medium, and small vessels.

Small-vessel vasculitis predominantly involves microscopic blood vessels with absent or insufficient immune deposits; its association with circulating autoantibodies to neutrophils (ANCA) has led to these conditions being grouped together as ANCA-associated vasculitis (AAV) (2, 3).

ANCA-associated vasculitis includes microscopic polyangiitis (MPA), granulomatosis with polyangiitis (GPA) renamed from Wegener’s granulomatosis, and eosinophilic granulomatosis with polyangiitis (EGPA), renamed from Churg–Strauss syndrome (4).

Microscopic polyangiitis is associated with proteinase 3 (PR3)-ANCA in 26% of cases and with myeloperoxidase (MPO)-ANCA in 58% of cases (5), while GPA is characterized by PR3-ANCA in 66% of patients and MPO-ANCA in 24% of patients (6).

Interestingly, some patients do not present ANCA; in particular, in EGPA more than an half are ANCA-negative (7).

ANCA-associated vasculitis has an approximate prevalence of 200 per million and an annual incidence of 20 per million.

The causes of AAV are unknown; it can be considered a complex disease, because multiple genetic factors, combined with yet unknown environmental factors, are able to influence its susceptibility (8, 9).

In addition, different phenotypes are influenced by several triggers and environmental factors (10).

Ethnicity affects the type of AAV, with Chinese and Japanese populations having more MPA and less GPA and EGPA than Caucasian, and the latter more than the African; geographical disparities in AAV have been found, with GPA and EGPA more common in colder climates. In Europe, GPA is more frequent in the north, and the reverse tendency has been found for MPA (11).

Similar treatment strategies used in trials involving patients with AAV, considered GPA and MPA as a single disease spectrum (6), hence the hypothesis that future genetic studies should group GPA and MPA together (12).

## Familial AAV Cases

The etiology of AAV theoretically involves interaction between genetics and environmental factors; however, knowledge about whether AAV clusters in families is poor.

Family recurrence is a strong indicator of a possible heritability in multifactorial disorders (13). Furthermore, information on the possible heritability of AAV is of clinical importance, because family members often want to know whether AAV increases their closest relatives at increased risk.

Familial AAV cases have been described but a putative hereditary factor has never been demonstrated.

The presence of EGPA in a family was described in two sisters with atopic-type bronchial asthma and negative perinuclear-ANCA results. The human leukocyte antigen (HLA) complex and six siblings were investigated, but the authors did not found a clear pattern of heritability (14).

An interesting study investigated the risk of GPA in relatives of patients with GPA, because several GPA families have been described (15). Using Swedish nationwide registers, the authors compared the occurrence of GPA among 6,670 first-degree relatives and 428 spouses of 1,944 Swedish patients with GPA, with the occurrence among 68,994 first-degree relatives and 4,812 spouses of 19,655 control subjects.

Granulomatosis with polyangiitis was present in 2/6,670 first-degree relatives of patients and in 13/68,994 first-degree relatives of the controls, with a relative risk of 1.56 (95% confidence interval 0.35–6.90).

The authors concluded that there is no evidence of an increase in familial risk of GPA, while it was demonstrated in other studies for other autoimmune disorders, such as inflammatory bowel disease (IBD), systemic lupus erythematosus (SLE), and multiple sclerosis (MS).

In several other cases, the familial members were affected by AAV and another type of vasculitis, underlying the possibility of common genetic triggers at the basis of vasculitis. In a first case, polyarteritis nodosa and GPA were observed in several members of two different families. The authors concluded that genetic factors are important but not sufficient to express clinical features of these diseases (16).

In another reported case, a man was affected by EGPA and his son by GPA (occurred 5 years later). These two patients lived together in Northern Italy and shared the HLA haplotype A*03-B*07-C*w07-DRB1*0404-DQB1*0302, a putative marker of autoimmunity (17). Recently, in an Indoasian family, three members of the same family with GPA who share the allele HLA-DPB1*04:01 were described, furthermore demonstrating the HLA locus is involved in AAV genesis (18).

## Pre-GWAS Association Studies in AAV

### Non-HLA associations

The classical approach for genetic studies applied to AAV is the case–control strategy, performed investigating candidate-gene polymorphisms. The dimension of the population, the ethnicity, the number of the investigated polymorphisms and the allele frequencies mainly determine the power of a study. The non-HLA single-nucleotide polymorphism (SNP) markers associated with AAV may contribute to the total variance in susceptibility, in addition to the HLA genes. The pre-GWAS non-HLA association studies are listed in Table 1.

**Table 1:** **Positive genetic association studies in anti-neutrophil cytoplasmic antibody-associated systemic vasculitis**.

Gene and variation	Population	Cases	Controls	OR	*P*-value	Other associated autoimmune diseases	Reference
*AAT (Z allele)*	German	79 GPA	752	3.8^a^	<0.0001	CD	(41)
*AAT (Z allele)*	Swedish	88 (66 GPA)	No controls	6.0	<0.0001	CD	(40)
*CD18 (Ava*II*)*	German	31 MPO^+^	120	2.56	<0.005	/	(44)
*CD40 (rs4810485)*	European Descent	895 GPA	1976	0.81	0.002	AT, T1DM, IBD, PS, MS, RA, and SLE	(90)
*CTLA4 (rs231735)*	European Descent	895 GPA	1976	0.82	0.001	RA	(90)
*CTLA (AT)86 repeat*	European Descent	117 GPA	123	Not available	0.0005	/	(40)
*CTLA4 (rs3087243)*	European Descent	895 GPA	1976	0.79	0.000098	GD, RA, T1DM, AITDs, and HA	(90)
*CTLA4 (rs3087243)*	British	641 (GPA, MPA, and EGPA)	9,115	1.19	6.4 × 10^−3^	GD, RA, T1DM, AITDs, and HT	(30)
HLA-DPB1*0401	German	282 GPA	380	2.47	6.4 × 10^−8^	/	(45)
HLA-DQB1*0303	Japanese	50 MPA	77	2:35	0.017	ACLT, SSc	(50)
*HLA-DQw7*	British	34 GPA, 25 MPA	1,103	2.9	<0.0025	/	(47)
*HLA-DR3*	British	34 GPA, 25 MPA	1,103	0.31	<0.01	/	(47)
*HLA-DR4*	Dutch	241 GPA, 30 MPA, 12 EGPA	9,872	1.7	<0.0001	/	(48)
*HLA-DR6*	Dutch	241 GPA, 30 MPA, 12 EGPA	9,872	0.3	<0.0001	/	(48)
HLA-DRB1*04	German	102 EGPA	341	1.86	0.0028	RA, GCA, AD	(52)
HLA-DRB1*07	Italian	48 EGPA	350	2:42	0.0042	SLE, AD	(51)
HLA-DRB1*07	German	102 EGPA	341	1.57	0.046	SLE, AD	(52)
HLA-DRB1*09	European Descent	76 GPA	4,039	4.00a	0.005	/	(49)
HLA-DRB1*0901-DQB1*0303	Japanese	50 MPA	77	2:44	0.0037	GD, MG, T1DM, RA, and SLE	(50)
HLA-DRB1*13	German	102 EGPA	341	0.50	0.019	Early childhood, MG	(52)
*HLA-DRB3*	Italian	48 EGPA	350	0:54	0.028	childhood ALL	(51)
*HLA-DRB3*	German	102 EGPA	341	0.61	0.004	childhood ALL	(52)
*HLA-DRB4*	Italian	48 EGPA	350	2:49	0.00023	HT	(51)
*HLA-DRB4*	German	102 EGPA	341	1.87	0.0002	HT	(52)
*IL-10*	Swedish	32 GPA	109	/	<0.05	SLE, SSc, KD, PM, DM, PV, UC, SS, GD, MG, PS, and ALPS.	(39)
*IL-10 haplotype*	German	103 EGPA	507	2:16	0.0003	SLE, SSc, KD, PM, DM, PV, UC, SS, GD, MG, PS, and ALPS.	(38)
*IL2RA rs41295061*	British	675 (GPA, MPA and EGPA)	8,936	0.77	0.012	T1DM, SLE	(30)
*PRTN3 -564G*	German	66 GPA	106	0.5	<0.01	/	(44)
*PTPN22 (rs2476601)*	European Descent	895 GPA	1976	1.35	0.002	CD, PS, RA, SLE, T1DM, GD, HT, and some forms of JA	(90)
*PTPN22 (rs2476601)*	German	199 GPA	399	1.8	0.002	CD, PS, RA, SLE, T1DM, GD, HT, and some forms of JA	(46)
*PTPN22 (rs2476601)*	Italian	143 GPA, 102 MPA, and 99 EGPA	945	1.91 GPA; 2.31 GPA ANCA^+^	0.005 GPA; 0.00012 GPA ANCA^+^	CD, PS, RA, SLE, T1DM, GD, HT, and some forms of JA	(31)
*PTPN22 (rs2476601)*	British	641 (GPA, MPA and EGPA)	9,115	1.4	1.4 × 10^−4^	CD, PS, RA, SLE, T1DM, GD, HT, and some forms of JA	(30)
*TLR9 3-SNP haplotype*	German, Dutch, British	646 GPA, 164 EGPA, and 53 MPA German AAV; 273 GPA, 53 EGPA and 100 MPA Dutch and British AAV cases	1898	1.64	0.000044	IBD	(37)
*FCGR3B CNVs number high*	British	556 (GPA, MPA and EGPA)	286	/	1 × 10^−8^	RA	(57)
*FCGR3B CNVs number low*	British	80 GPA	190	/	0.003	SLE	(69)
*FCGR3B CNVs number low*	British	76 MPA	190	/	0.0003	SLE	(69)
*FCGR3B CNVs number low*	French	84 GPA	181	/	0.0001	SLE	(69)
*DEFB4 CNVs number high*	Chinese	112 AAV (ANCA^+^ and biopsy proven necrotizing glomerulonephritis)	523	/	0.009	SLE, PS, COPD, IBD, HIV infection, and SAP	(65)

*ANCA, autoantibodies to neutrophils; OR, odds-ratio; CNVs, copy number variations; GPA, granulomatosis with poliangiitis; MPA, microscopic poliangiitis; EGPA, eosinophilic granulomatosis with poliangiitis; CD, Crohn’s disease; T1DM, diabetes mellitus type1; CD, celiac disease; RA, rheumatoid arthritis; MS, multiple sclerosis; pSS, primary sicca syndrome; AT, autoimmune thyroiditis; IBD, inflammatory bowel disease; AITD, autoimmune thyroid disease; GB, Guillain–Barrè syndrome; MG, myasthenia gravis; ACLT, autoimmune chronic lymphocytic thyroiditis; SSc, systemic sclerosis; GCA, giant cell arteritis; ACPAs, anti-citrullinated protein antibodies; KD, Kawasaki disease; DM, dermatomyositis; PV, pemphigus vulgaris; UC, ulcerative colitis; SS, Sjogren’s syndrome; ALPS, autoimmune lymphoproliferative syndrome; SAP, severe acute pancreatitis; GD, Grave’s disease; COPD, chronic obstructive pulmonary disease; PS, psoriasis; HT, Hashimoto thyroiditis; JA, juvenile arthritis; AD, atopy*.

#### Protein tyrosine phosphatase, non-receptor type 22 (lymphoid) (PTPN22)

The rs2476601 PTPN22 SNP is linked with several autoimmune diseases. The first associations between rs2476601 SNP of PTPN22 gene and autoimmune diseases were demonstrated with type 1 diabetes (T1D) (19) and rheumatoid arthritis (RA) (20); after that, the finding expanded to other autoimmune diseases, enhancing the importance of signaling thresholds in disease susceptibility; even if it is difficult to determine how threshold effects change disease risk. PTPN22 gene encodes for an intracellular tyrosine phosphatase that dephosphorylates activating tyrosines, as well as other molecules involved in TCR signaling. The transgenic mouse homolog of PTPN22 shows hyperactive T cells (21). In human beings, the PTPN22 gene can present a SNP at the codon 620 (Arginine to Tryptophan), a change able to destroy the binding of PTPN22 with the binding partner, the intracellular tyrosine kinase, Csk. However, the consequence of this for T cell activation thresholds remains difficult to interpret. The first studies of PTPN22 risk allele carriers an increased phosphatase activity with reductions in TCR and BCR signaling (22). Recent studies have supported the hypothesis that the 620W allele acts as the knock-out mouse, with loss of function leading to hyper-responsiveness. The lack of PTPN22 results in changes in T cell populations in mice, particularly an expansion of T reg cells (23). In addition to T cell influence, also B cell receptor signaling appears to be affected by PTPN22 SNP (24). The protein Csk has recently been demonstrated as a susceptibility gene for several autoimmune diseases such as Celiac disease (CD) (25), Scleroderma (26) and SLE (27). Thus, both PTPN22 and Csk may influence disease risk affecting the B cell receptor repertoire development. Taken together, these data suggest that groups of genes are able to influence receptor signaling thresholds; PTPN22 and Csk, in fact, may influence the risk of autoimmunity influencing the autoimmune repertoire selection and the abundance of regulatory cell subsets. (28). It is highly probable that different mechanisms explain the genetic associations for every autoimmune disease.

The first study of the rs2476601 SNP in AAV has been performed in a German GPA cohort, demonstrating a statistical association; interestingly, stratifying the GPA patients in ANCA-positive and ANCA-negative, the association was stronger in the ANCA-positive subgroup (29). Later, the result was confirmed in British GPA and MPA patients (30) and Italian AAV cases (31). The Italian study confirmed the strong association with the ANCA-positive subset, while suggested that EGPA is not associated with the PTPN22 SNP.

#### Cytotoxic T lymphocyte associated antigen-4

Cytotoxic T lymphocyte associated antigen-4 (CTLA-4) is a regulatory molecule expressed on T cells that plays a major role in peripheral tolerance and inhibiting T cell activation (32).

CTLA-4^−/−^ knock-out mice show a phenotype rapidly developing in lymphoproliferative disease with multiorgan lymphocytic infiltrations, tissue destruction, splenomegaly, lymphadenopathy, and elevated serum immunoglobulin with early lethality, demonstrating a predominant role in suppressing T cell function (33). It is likely that defective CTLA-4 expression and function are associated with autoimmune diseases. Furthermore, CTLA-4 activity influences a negative regulator of both cellular and humoral responses and mediates antigen-specific apoptosis.

Negative signaling of CTLA-4 has an active role in the regulation of autoreactive T cells, and disturbance of the normal control of the CTLA-4 may cause the pathogenesis of autoimmune diseases.

The CTLA-4 genetic polymorphisms alter activity influencing the CTLA-4 gene expression.

An AAV associated polymorphism is the allele (named allele 86) of the CTLA-4 microsatellite polymorphism (AT)n, which maps in the 3′-untranslated region (3′-UTR) of exon 3. Individuals with long CTLA-4 (AT)n repeat alleles show hyper-reactive T cells.

The CTLA-4 (AT)n 86 allele has been demonstrated to be important for maintenance of normal levels of CTLA-4 expression and seems to balance T cell activation/inhibition. A study in European descent GPA patients (34) confirmed findings from a Scandinavian cohort in which a positive association with the long alleles of (AT)n in the CTLA-4 3′-UTR was demonstrated. Evidences of a blockade of T cell costimulation using CTLA-4Ig seems to be a potential therapeutic intervention, alternative to standard treatment with immunosuppressive agents. In a recent open-label trial of 20 patients with non-severe relapsing GPA, the safety and efficacy of abatacept were investigated. The authors concluded abatacept was well tolerated and an high frequency of disease remission and prednisone discontinuation were observed. This study demonstrated anti-CTLA4 may be an option in the treatment of some subgroups of AAV (35).

A second polymorphism in CTLA-4 gene investigated in AAV is the SNP rs3087243. A large British study found the minor allele (A) of this SNP is protective for AAV (30).

#### Toll-like receptors

Evidences are emerging about the role of Toll-like receptors (TLRs) in the development of the autoimmune diseases (36).

Toll-like receptors are a family of innate receptors whose specificities are predetermined in the germline. Therefore, TLRs have evolved to recognize conserved features of microbes. Viruses typically lack the conserved features common to other pathogen classes, so the innate immune system has evolved to recognize viral nucleic acid as a hallmark of viral infection.

TLR9 signaling may be involved in disease pathology, favoring models of infectious agents triggering AAV development. Unmethylated CpG motifs within bacterial nucleic acid were the first ligands identified to activate TLR9. Since then, many DNA viruses have also been shown to activate this TLR, including those from the herpesvirus and adenovirus. Both α- and β-herpes viruses have genomes that are rich in CpG motifs. The contribution of TLR9 recognition of these viruses has clearly been reported *in vitro* (37).

In order to investigate the genetic contribution of TLR9 on the susceptibility and clinical manifestation of AAV, four SNPs in TLR9 have been genotyped in 863 German AAV cases and 1344 healthy controls (38). In the replication step, significant results were investigated in a cohort of 426 Dutch and British AAV cases. Interestingly, in GPA patients the association with genotypes and haplotypes was predisposing (OR >1), while in MPA was protective (OR <1). When cases were stratified according to ANCA status rather than to clinical entity, the association was confirmed; in fact, the results showed a strong overall difference in TLR9 allele/haplotype frequencies between PR3-ANCA and MPO-ANCA cases. These results confirmed the findings of the EVGC GWAS about the genetically differences between PR3-ANCA and MPO-ANCA AAV.

#### Interleukin-10

Interleukin-10 (IL-10), which was originally named cytokine synthesis inhibitory factor, is a cytokine that is produced by type T-helper cells. IL-10 presents anti-inflammatory properties, with the inhibition of immune mediator secretion, antigen presentation, and phagocytosis.

A study investigated the presence of IL10 gene polymorphisms in EGPA and GPA patients of European ancestry (39). Three SNPs in the IL10 gene promoter (IL10-3575, IL10-1082, and IL10-592) were analyzed in 403 GPA patients and 103 EGPA patients, compared with 507 German controls. The IL10-3575/-1082/-592 TAC haplotype, part of the extended ancient haplotype IL10.2, was highly significantly associated with ANCA-negative EGPA, providing further evidences that AAVs have distinct genetic backgrounds. Other studies of the IL-10 SNPs were performed both in German and Swedish population, suggesting the IL-10 may influence the production of autoantibodies (40).

#### SERPINA1

The SERPINA1 gene encodes for α1-antitrypsin, a neutral serine protease inhibitor, which includes proteinase 3; this is the major inhibitor of PR3 activity, and is supposed to limit the damage to the local tissues. This mechanism may be crucial in AAV, because the decreased function of α1-antitrypsin potentially results in persistence of PR3 in inflammatory tissue, with the final consequence of ANCA generation.

The SERPINA1 gene is highly polymorphic; the Z allele (Glu342Lys) in α1-antitrypsin (AAT) deficiency is a combined deficiency and dysfunctional allele.

Several studies investigated the role of the Z allele in AAV, showing that heterozygous patients for the Z variant of the SERPINA1 gene have an increased risk than the general population of developing GPA (41, 42).

#### PRTN3

PRTN3 gene encodes for proteinase 3 (PR3) protein, a neutrophil intracellular protease that is the main antigen of ANCA autoantibodies; it is located on the plasma membrane of a subset of neutrophils. The rate of this membrane PR3-positive neutrophils subset is characteristic of an individual, and this semms to be genetically determined (43).

In GPA patients, there is an increased number of PR3-positive neutrophils and an elevated level of PRTN3 expression compared with controls. Increased levels of PRTN3 expression are associated with an elevated risk of relapse and with an increased relapse rate; this supports the idea that PR3 expression on the membrane of neutrophils plays a role in the pathophysiology of PR3-AAV (44).

The promoter and coding regions of the PRTN3 gene were sequenced searching for genetic variants in 79 GPA patients and 129 healthy controls. Seven SNPs, one amino acid change (Val119Ile), one 84 bp insertion/deletion, and a microsatellite were found. An association with GPA was demonstrated for the A-564G polymorphism in the PRTN3 promoter; this variation affects a candidate transcription factor-binding site. The authors suggested the overexpression of PRTN3, might predispose the patient to the development of AAV (45).

### HLA associations

The HLA region, located on chromosome 6p21 and extended for 7.6 Mb, is the most dense gene region within the human genome encoding more than 250 expressed loci including several key immune response genes. The region can be subdivided in class I, II, and III regions and contains the highest number of polymorphisms and the most dense linkage disequilibrium in the human genome. In the last 50 years, numerous associations between the HLA region and autoimmune disease have been demonstrated. The association between HLA and AAV has been largely investigated, for different populations and with several methods. The pre-GWAS HLA association studies are listed in Table 1.

The most studied AAV has been GPA, with demonstrated association for DPB1*0401 (46, 47), DQw7 (48), and different DRB1 genes, some with protective effects [DRB1*03 (47), DRB1*06 (48)], other with causative effects [DRB1*04 (49), DRB1*09 (50)]; in some cases the populations were small and the results have not been replicated.

Microscopic polyangiitis has been investigated in few studies, mainly because the incidence of the disease is smaller than GPA. Associations were demonstrated with DRB1*03 (48) and DRB1*06 (49) (protective effects), while DRB1*04 (49) and the haplotype DRB1*0901-DQB1*0303 (51) have causative effects.

Finally, EGPA has been mainly investigated in Italian (52) and German (53) populations, with the same results. The HLA genes DRB4 and DRB1*07 have causative effects while DRB3 and DRB1*13 with protective effects. Furthermore, the first study stratified EGPA in the two major clinical subsets (54), ANCA-negative, with organ damage mainly mediated by tissue eosinophilic infiltration and ANCA-positive, with features of small-vessel vasculitis; analyzing the HLA-DRB4 in patients categorized by different numbers of vasculitic manifestations (purpura, alveolar hemorrhage, mononeuritis multiplex, rapidly progressive glomerulonephritis, and constitutional symptoms) suggested that the gene frequency correlates with the different number of vasculitis symptoms.

All the studies examined relative small population, and need to be confirmed in large studies with focused GWAS. Furthermore, the different associations between GPA, MPA, and EGPA demonstrate the three AAV have a different genetic background.

## GWAS AAV Genetics

The main aim of a GWAS is to identify common genetic variation associated with well phenotyped disease in a non-biased method. Inherent to the properties of association-based statistics, the premise for conducting GWAS is based on the assumption that disease risk is associated with relatively common variants with a minor allele frequency greater than 2–5%. The goal is to identify frequency differences in polymorphisms between cases and controls. As a result, the study analyses a large sample size with a strong statistical correction in order to avoid spurious associations due to false-positive findings. The occurrence of linkage disequilibrium is used because not all the polymorphisms can be genotyped to detect an association. The larger the population studied for a disease, the more associated variants can be discovered (55).

To date, two different GWASs have been performed in AAV. The first one was performed by the European Vasculitis Genetic Consortium (EVGC) (56) and the second one by the VCRC (Vasculitis Clinical Research Center) (57). The two GWASs differed for the study design.

In 2012, the EVGC performed the first GWAS of AAV (ANCA-positive GPA and MPA) in order to identify common or specific genetic-risk factors.

The clinical diagnosis of AAV was evaluated according to the European Medicines Agency algorithm, supported by either a positive ANCA assay or a diagnostic biopsy.

The EVGC GWAS was performed in 1233 UK patients with AAV and 5884 controls (from the Wellcome Trust Case–Control Consortium) and was replicated in 1454 Northern European case patients and 1666 controls. A total of 2687 AAV patients of European ancestry and 7650 controls were analyzed. The main results of the EVGC GWAS are listed in Table 2.

**Table 2:** **Associations of single-nucleotide polymorphisms (SNPs) and antineutrophil cytoplasmic antibody (ANCA)-associated vasculitis (GPA^+^ MPA), According to Cohort**.

				Combined cohort (*N* = 2267 case patients, 6858 controls)	
Gene	Chromosome	SNP (Minor Allele)	Nucleotide change	OR	*P*-value
**SNPs WITH GENOME-WIDE SIGNIFICANCE IN COMBINED COHORT**					
*HLA-DP*	6	rs3117242	A > G	3.67	1.5 × 10^−71^
*HLA-DQ*	6	rs5000634	A > G	0.80	2.9 × 10^−9^
*COL11A2*	6	rs3130233	A > G	1.51	7.8 × 10^−15^
*COL11A2*	6	rs3117016	A > G	1.83	6.4 × 10^−24^
*SERPINA1*	14	rs7151526	A > C	0.59	2.4 × 10^−9^

				**Clinical syndrome**	
				**GPA vs. control (*N* = 1683 vs. 6858)**	**MPA vs. control (*N* = 489 vs. 6858)**
**Gene**	**Chromosome**	**SNP**	**Nucleotide change**	**OR**	***P*-value**

**ASSOCIATIONS OF SNPs AND ANCA – ASSOCIATED VASCULITIS, ACCORDING TO CLINICAL AND ANCA SUBGROUPS**					
*HLA-DP*	6	rs3117242	A > G	5.39	3.1 × 10^−85^
*HLA-DQ*	6	rs5000634	A > G	0.83	2.2 × 10^−6^
*ARHGAP18*	6	rs1705767	A > C	0.78	3.3 × 10^−7^
*SERPINA1*	14	rs7151526	A > C	0.54	4.4 × 10^−10^
*PRTN3*	19	rs62132295	A > G	0.78	2.6 × 10^−5^
*MOSPD2*	X	rs6628825	A > G	0.80	2.6 × 10^−6^

				**ANCA specificity**			
				**Proteinase 3 vs. control (*N* = 1521 vs. 6858)**		**Myeloperoxidase vs. control (*N* = 556 vs. 6858)**	
							
**Gene**	**Chromosome**	**SNP**	**Nucleotide change**	**OR**	***P*-value**	**OR**	***P*-value**
*HLA-DP*	6	rs3117242	A > G	7.03	6.2 × 10^−89^	1.55	3.2 × 10^−2^
*HLA-DQ*	6	rs5000634	A > G	0.86	3.3 × 10^−5^	0.65	2.1 × 10^−8^
*ARHGAP18*	6	rs1705767	A > C	0.73	5.2 × 10^−8^	0.87	1.0 × 10^−2^
*SERPINA1*	14	rs7151526	A > C	0.53	5.6 × 10^−12^	0.84	2.8 × 10^−1^
*PRTN3*	19	rs62132295	A > G	0.73	2.6 × 10^−7^	1.10	2.2 × 10^−1^
*MOSPD2*	X	rs6628825	A > G	0.77	6.1 × 10^−7^	0.86	6.3 × 10^−1^

*GPA, granulomatosis with poliangiitis, MPA, microscopic poliangiitis, EGPA, eosinophilic granulomatosis with poliangiitis*.

In order to confirm the GWAS data, 156 SNPs were genotyped in a replication cohort of 1454 AAV patients and 1666 controls.

In addition to the SNPs genotyped in the chip, three additional SNPs were included: the proteinase 3 gene (PRTN3), which is a major ANCA autoantigen, the interleukin-2-receptor alpha gene (IL2RA) and the protein tyrosine phosphatase, non-receptor type 22 gene (PTPN22) because were found to be associated with AAV in previous studies.

Combined analysis of the discovery and replication cohorts showed that these SNPs were statistically significant. Three of them mapped in the HLA locus, with the most significant, which maps in the HLA-DPB1 gene, while a fourth associated SNP maps in the SERPINA1 locus at 14q32; this demonstrates that the susceptibility loci are located bot on HLA and in non-HLA regions. The SERPINA1 gene encodes for the protein α1-antitrypsin, a neutral serine protease inhibitor, which includes proteinase 3. Associations with the rare Z (null) allele of SERPINA1 have been described in previous studies, but the significance were low (58).

The SNP in the SERPINA1 locus is in linkage disequilibrium with the Z allele, suggesting that the causal variant at the SERPINA locus is either the Z allele of SERPINA1 or is in close linkage disequilibrium with it.

In order to investigate whether GPA and MPA are genetically distinct clinical diseases, a further statistical analysis of the discovery cohort was subdivided in the two diseases according with clinical data comparing the seven most significant SNP associated with AAV in the two diseases. Interestingly, the three HLA and SERPINA1 SNPs differed significantly between GPA and MPA, with almost all the association related to GPA.

Finally, a comparison between the subgroups of patients with PR3-ANCA and with MPO-ANCA, evidenced significant differences at the HLA, SERPINA1, and PRTN3 loci, demonstrating a genetic association with PR3-ANCA but not with MPO-ANCA.

In the GPA subgroup, the associations were found only in PR3-ANCA patients, not in MPO-ANCA patients. Within the MPA group, the findings were the same: an association of PR3-ANCA and the HLA-DP and SERPINA1 SNPs. The most significant SNP associated with AAV in non-MHC regions was the rs7151526, both in GPA and PR3-ANCA subgroups; the Z allele of this SNP maps in the SERPINA1–SERPINA11 locus at 14q32.

The major finding of the EVGC GWAS was that the strongest association of all these genetic polymorphisms appeared to be related with ANCA specificity rather than the clinically defined syndromes. The genetic background with autoantibody specificity evidenced in this study could impact the clinical classifications of GPA and MPA and therapeutic options (59).

Further GWAS with large ANCA-negative AAV are needed in order to find the genetic basis of ANCA-negative GPA and MPA.

A second GWAS was performed by the VCRC in 492 GPA patients (white subject of European descent) and 1,506 healthy controls; the data were replicated in a second cohort of 528 GPA patients and 1,228 controls. The main results of the VCRC GWAS are listed in Table 3. Association analysis of the two combined cohort (750 patients and 1,820 controls) showed that the strongest association mapped in the HLA region, with the SNP rs9277554 (HLA-DPB1 gene, *P* = 1.92 × 10^−50^), and rs9277341 (HLA-DPA1 gene, *P* = 2.18 × 10^−39^). An independent single SNP, rs26595, showed also association with GPA in the combined cohort (*P* = 2.09 × 10^−8^). This SNP maps on chromosome 5 in proximity of the SEMA6A gene, which encodes for a protein called semaphorin 6A. Semaphorins are involved in many physiologic processes, such as vasculogenesis, cardiogenesis, osteoclastogenesis, tumor metastasis, and immune regulation (60, 61). The role of SEMA6A in GPA is still unclear; a possible theory explains that semaphorins have an important role in the immune response in autoimmune and allergic disorders (62). The association between the rs26595 SNP of the SEM6A6 gene and GPA was recently investigated in 879 GPA patients, 150 MPA patients and 191 EGPA patients, compared with 1,376 healthy control subjects (63). All of the subjects were white Europeans. ANCA status was available for 90% of the patients. The authors did not observe any statistically significantly different allele frequencies between AAV patients and controls, not confirming the findings from the initial VCRC GWAS. Between the two studies the approaches of analysis is different, raising the need for further genetic association studies in GPA.

**Table 3:** **Results of genome-wide association (stage 1) and replication (stage 2a) analyses of GPA association**.

				Combined analysis (*n* = 750 case patients, *n* = 1820 controls)	
Gene	Chromosome	SNP	Nucleotide change	OR	*P*-value
*HLA-DPB1*	6	rs9277554	T > C	0.24	1.92 × 10^−50^
*HLA-DPA1*	6	rs9277341	C > T	0.33	2.18 × 10^−39^
**Non-HLA region**
*WSCD1*	17	rs7503953	A > C	1.50	1.93 × 10^−7^
*COBL*	7	rs1949829	T > C	1.78	4.19 × 10^−7^
*CCDC86*	11	rs595018	A > G	1.46	1.60 × 10^−7^
*DCTD*	4	rs4862110	C > T	1.44	2.14 × 10^−6^

				**Combined analysis (*n* = 987 case patients, *n* = 2731 controls)**	

*SEMA6A*	5	rs26595	C > T	0.74	2.09 × 10^−8^

				**c-ANCA-positive patients vs. controls (patients, *n* = 578; controls, *n* = 1,820)**	
**Gene**	**Chromosome**	**SNP**	**Nucleotide change**	**OR**	***P*-value**

**VCRC GWAS: associations of MHC Loci with c-ANCA-positive GPA**					
*HLA-DPB1*	6	rs9277554	T > C	0.16	4.7 × 10^−57^
*HLA-DPA1*	6	rs9277341	C > T	0.27	2.30 × 10^−42^

*GPA, granulomatosis with poliangiitis; SNP, single-nucleotide polymorphism; OR, odds-ratio; VCRC, Vasculitis Clinical Research Consortium; ANCA, autoantibodies to neutrophils*.

SERPINA1 has been the only non-MHC locus associated with AAV. However, the SNP strongly associated in that study (rs7151526) is located 557 bp from rs1956707 and was not independently associated in that population; the reason may be the two SNPs are not in linkage disequilibrium.

The potential HLA associations to specific GPA subsets were also investigated. Comparisons of the HLA risk allele frequencies between the PR3-cANCA positive subgroup (88% of cases) and the ANCA-negative subgroup showed that the associations with HLA–DPB1 (rs9277554, *P* = 4.7 × 10^−57^) and HLA–DPA1 (rs9277341, *P* = 2.30 × 10^−42^) were caused by the PR3-cANCA positive group, a finding, which confirms previous data suggesting genetic differences between ANCA-positive and ANCA-negative GPA.

The differences between the two GWAS results may be explained to different factors, such as phenotypes in the patient cohorts (i.e., ANCA-positive GPA and MPA in the EVGC versus ANCA-positive and -negative GPA in VCRC) and in the genetic and statistical approaches.

Further genetic studies of additional cohorts are needed to clarify the AAV genetic background. The two different GWAS have identified SERPINA1, SEMA6A, and HLA loci as significant factors related to AAV risk and demonstrate that the strongest association is linked to HLA–DPB1*04. Although the results must be confirmed in further studies, they highlight that MPA and GPA might be considered two distinct clinical entities, and that ANCA specificity might be segregated in different subgroups, probably better than the distinction actually made between GPA and MPA; as well, the autoantigen PR3 might be a direct player/trigger in the pathophysiology of GPA.

## Copy Number Variations

Copy number variations (CNVs) have been defined by the presence of variable copies (deleted or duplicated) of genomic regions of 1 kb or more in different individuals; CNVs represent an important source of genetic variation in the human genome (64).

Approximately 360 Mb pairs (12% of the human genome is represented by CNVs), with a preponderance of smaller size rearrangements (<20 kb). The genomic regions with these CNVs contain hundreds of genes and other functional elements, and many CNVs have a population frequency higher than 1% [copy number polymorphisms (65)].

CNVs can change the gene dosage of particular gene, influencing the susceptibility to a specific disease.

Immunological genes are frequently involved in CNVs, perhaps because this represents a mechanism to expand the recognition repertoire. To date, a low number of studies have been performed in autoimmune diseases, even if immunological genes have the potential to be affected by CNVs.

A first demonstration that CNVs are linked with AAV has been found in a Chinese population; in a study of 112 AAV (ANCA positive and biopsy proven necrotizing glomerulonephritis) patients and 523 controls, an association of CNVs in the DEFB4 gene has been statistically demonstrated (65). This gene belongs to the β-defensin family genes, underlying a possible mechanism of susceptibility in some inflammatory disorders, such as psoriasis (Ps), chronic obstructive pulmonary disease, IBD, HIV infection, and severe acute pancreatitis. Furthermore, at mRNA level, there is a correlation between CNVs and β-defensin gene expression (66).

Defensins show a vast spectrum of anti-microbial activity, representing an important factor in the first defense against micro-organisms and connect the innate and adaptive immune system. These functions are performed with the production of chemotactic factors inducing pro-inflammatory cytokines.

A further CNV analysis in AAV has been performed studying the FCGR3B gene.

Low-affinity Fc-receptors (FcgR) play a crucial role in the initiation and regulation of the antibody-mediated immune response, linking humoral, and cellular immunity. These receptors are able to recognize the constant domain of IgG and are involved in the mobilization of macrophages, natural killer T-cells, and neutrophils to regions of immune complex deposition. FCGRs also play an important role in the recognition and clearance of immune complexes, and as modulators of B-cell activity. In particular, FCGR3B CNV has been investigated in risk of autoimmunity (67).

Association between FCGR3B CNV and a number of autoimmune disorders has been reported in European populations, including SLE (68), RA (69), and in Japanese population for ulcerative colitis (64).

In the investigation of the association between FCGR3B CNV and AAV, it is noteworthy that the approach they used for statistical analysis; in a previous paper, an association with AAV and low FCGR3B CNV by Quantitative Polymerase Chain Reaction was utilized (70), but analyzing a UK Caucasian vasculitis cohort with a triple paralog ratio test the association was not confirmed, suggesting that the initial finding may have been a false-positive resulting deriving from the lack of robustness in the qPCR assay (71).

## Epigenetics

Epigenetics can be defined as those mechanisms that cause stable changes of gene expression without influencing the primary nucleotide sequence; it might explain why human body cells have highly specific phenotypes, even though they all carry an identical genome (72).

Epigenetic mechanisms are particularly important for autoimmunity, since the expression of pro-inflammatory genes (like TNF-α) is regulated at the chromatin level.

Little is known about epigenetics in AAV; in 2010 a study investigated the role of epigenetics in AAV (73), this was based on the concept that the development of AAV basically needs two things: a dysregulation in immune response able to produce autoantibodies directed to PR3 and MPO and high expression of these major ANCA autoantigens in mature neutrophils. When the autoantibodies find the aberrantly autoantigens presented on neutrophils they can stimulate activation of neutrophils causing vascular inflammation. The data suggested that epigenetic modifications (mainly DNA methylation and histone modification) associated with gene silencing are able to influence ANCA autoantigen encoding genes, potentially influencing the expression of PR3 and MPO in AAV patients, and suggesting that epigenetic events may be important in the development of autoimmunity.

This study and other investigations in autoimmune diseases suggest epigenetics globally affects these disorders and that the persistence of the epigenomic changes could lead to an aberrant gene expression, contributing to the perpetuation of the disease mechanisms.

## Pharmacogenetics

The pharmacogenetics is the study of the role of inherited and acquired genetic variation in drug response (74). These type of studies scans for candidate genetic variants of subjects based on the different type of response for a specific drug, in a case–controls design. Pharmacogenetics has the potential to predict response and/or toxicity based on genetic background and has the potential to personalize therapy. The aim of the pharmacogenetics is to personalize the specific theraphy, enhancing the response and minimizing the side effects of the chemotherapeutic drugs. In recent years, the development of monoclonal antibodies directed against a particular immunological protein has permitted the treatment of several autoimmune disorders [i.e., RA and AAV, autoimmune hemolytic anemia, T1D, Sjogren’s syndrome (Sj)]. One of these most used drugs is rituximab (RTX), a chimeric monoclonal antibody directed against the protein CD20. RTX is able to destroy normal and malignant B cells that present CD20 (primarily found on the surface of the B cells) on their surfaces; this is useful for the treatment of those diseases characterized by a high number of iperactive or dysfunctional B cells.

Unfortunately, RTX has several side effects, such as disability, cardiac arrest, syndrome of tumor lysis (which can cause acute renal failure), infections (Hepatitis B reactivation), progressive multifocal leukoencephalopathy, immune toxicity (with depletion of B cells), and pulmonary toxicity. Sometimes RTX can cause death. RTX acts with a mechanism mediated by the Fc portion, including apoptosis of CD20-positive cells, complement-dependent cytotoxicity (CDC), Fcg receptor (FcgR)-mediated mechanisms, antibody-dependent cellular cytotoxicity (ADCC), and phagocytosis. These combined effects can stimulate elimination of B cells with the generation of normal B cells developed from lymphoid stem cells (75).

Several polymorphisms able to influence the RTX activity have been identified. FCGR3A gene, which encodes the FcgRIIIa protein, presents the c.559 SNP, which can change the aminoacid in position p.158 of the FcgRIIIa, affecting the affinity for human IgG1 (76, 77); as a consequence, human IgG1 binds more strongly the NK cells homozygous for FCGR3A-158V than to NK cells homozygous for FCGR3A-158F.

Because FcgRIIIa is expressed only by monocytes/macrophages and NK cells (the main actors in ADCC), it has been postulated that patients homozygous for FCGR3A-158V show significantly better responses to RTX because they have enhanced ADCC activity compared with FCGR3A-158F carriers. This was confirmed in other studies using RTX (78, 79).

Weng and Levy (78) also showed that the FCGR2A-131H/R polymorphism significantly affects response and time to progression after RTX treatment, with better results for FCGR2A-131H homozygous patients. These results were not confirmed in other studies, that found no impact on response (80), suggesting the association may arise from linkage disequilibrium between FCGR3A/FCGR2A polymorphisms (81, 82).

In addition to the influence of FCGR2A and FCGR3A in RTX action, it is important to consider that the drug binds to aminoacids 170–173 and 182–185 on CD20 protein; these residues are physically close to each other for the creation of a disulfide bond between amino acids 167/183, creating a potential mechanism of different response to RTX based on CD20 mutations.

To date, there is no evidence that inherited mutations or polymorphisms of the CD20 gene (called MS4A1) influence the response to RTX (83).

Recently, an Italian group selected 132 patients with systemic autoimmune diseases treated with RTX.

Of these patients, 81 (61.4%) were SLE patients, 16 (12.1%) presented different inflammatory myopathies such as polymyositis and dermatomyositis, 13 (9.8%) were AAV patients (GPA, EGPA and MPA); the remaining 22 patients suffered of other systemic autoimmune diseases such as Sj, systemic sclerosis (SSc), or autoimmune hemolytic anemia.

Genotyping for FCGR3A-158F/V (rs396991) gene polymorphism was performed, in order to determine whether the SNP 158F/V in the FCGR3A gene, influences the RTX response in systemic autoimmune disease patients.

The type of drug response after RTX infusion was evaluated: 61% of the patients had a complete response, partial response 27 and 12% did not respond to the treatment. A statistically significant difference was observed in 158 V allele frequency between responder (38%) and non-responder (16%) patients (*P* = 0.01; OR = 3.24). RTX was also more effective in the 158 V allele carriers (94%) than in homozygous 158FF patients (81%): *P* = 0.02; OR = 3.96. This study shows that FCGR3A-158F/V SNP influences the response to RTX in autoimmune diseases, including AAV (results need to be replicated in a larger population, because the AAV population was small) (84).

Another drug used in the treatment of AAV is the cyclophosphamide (CP), a bifunctional DNA alkylating agent; this drug is used for the treatment of pediatric and adult malignancies, because it is characterized by anti-tumor activity in human beings (85). At low dosage, CP is a potent immunomodulatory used as second-line therapy (86). Like other chemotherapy, CP shows difference in efficacy and toxicity in patients. CP side effects include cardiotoxicity, nephrotoxicity, neurotoxicity, infertility, bladder toxicity, myelosuppression, and leukemogenesis. CP bioactivation and/or detoxification has demonstrated to be influenced by different SNPs; with the era of –omics, however, new methods can increase knowledge about the genetic background able to influence CP activity (87). Finally, genes found having pharmacokinetic and pharmacodynamic effects, require replication in larger and more diverse patient populations.

## Overlap with Other Autoimmune Diseases

Several HLA and non-HLA susceptibility variants appear to increase risk in autoimmune diseases, which share the same pathway even if the diseases are different. In recent years, candidate-gene studies in AAV have often investigated variants previously associated to other autoimmunity diseases.

The two most investigated genes, which are potentially associated with autoimmune diseases are CTLA-4 and PTPN22.

The CTLA-4 SNPs rs3087243 and rs231735 have been previously found to be associated with RA and T1D, and replicated in GPA (34, 88–90).

In 2012, a study examined the association between previously identified autoimmune disease susceptibility loci and GPA, trying to demonstrate shared genetic susceptibility variants (91). The study examined 431 GPA patients and 473 controls, all of European ancestry. Multiple SNPs were assessed to be associated with GPA, shared with other autoimmune diseases such as Crohn’s disease, T1D, SLE, RA, CD, and ulcerative colitis. SNPs in CTLA-4 gene were significantly associated with GPA in the single-marker meta-analysis [odds ratio (OR) = 0.79, *P* = 9.8 × 10^−5^]. The genetic-risk score for RA susceptibility markers was significantly associated with GPA (*P* = 5.1 × 10^−5^). The results may demonstrate that RA and GPA share a similar genetic background.

This study supports the findings of a previous epidemiologic study, which showed an increased frequency of RA among offspring of GPA patients (92). GPA and RA present several differences, such as the pulmonary and renal manifestations (not commonly observed in RA, but frequent in GPA); furthermore, inflammatory arthritis (characteristic of RA), is not present in every patients with GPA.

Interestingly, this meta-analysis showed that other loci different from CTLA-4 previously found to be associated with common autoimmune diseases were not statistically significantly associated with GPA in this study.

The PTPN22 rs2476601 (R620W) polymorphism has been found to be associated with GPA (29) and with multiple other autoimmune diseases (23).

Recently, a comprehensive meta-analysis (93) showed that PTPN22 C1858T SNP is strongly associated with nine autoimmune diseases: T1D, RA, SLE, Graves’ disease, Addison’s disease, AAV, juvenile idiopathic arthritis, myasthenia gravis and Graves’ disease with other concomitant autoimmune diseases, with the T allele increasing the risk of disease. The association suggests that is possible the presence of a common mechanism shared by these autoimmune diseases. Further investigation of these mechanisms may in the future shed light in the pathogenesis of the autoimmune diseases. The studies related to the PTPN22 R620W variant suggest that there is a correlation between the presence of autoantibodies and the development of self-reacting antibodies, with the PTPN22 620W, which may affect autoimmune response on several levels, including generation and activation of autoimmune cells. Based on this theory, the PTPN22 function in autoimmune disease manifestations seems to affect generation and activation of immune cells (93).

In conclusion, the sharing of a similar genetic background suggests that different autoimmune diseases may have similar pathogenic mechanisms, and the shared association with CTLA-4 and PTPN22 variants affects the threshold for activation or deactivation of autoreactive T cells.

Further GWAS results and other genomic approaches can increase the knowledge of the genetic background of AAV, searching for rare variants that would be not investigated in previous studies.

## Future Perspectives

In complex diseases, it is necessary to search new associated loci, but also replicate already known susceptibility variants. Novel demonstrated loci confer low increases in susceptibility to the disease, but they may evidence new pathways important in etiology or that may be crucial for pharmacogenetic applications. To date, the HLA complex still show the strongest association for several autoimmunity diseases (like AAV), but different non-HLA genes associated with autoimmunity are now under investigation.

There are now several methods available for investigating more deeply the genomic regions associated with autoimmunity.

### Immunochip

In recents years, Illumina produced ImmunoChip genotyping SNPs microarray, which allows immunogenetics studies of the major inflammatory and autoimmune diseases, mapping with high-density previous established GWAS significant loci. The Immunochip contains 196,524 polymorphisms, with 195,806 SNPs and 718 small insertion/deletions, enabling cheap fine mapping of loci for common and rare variants. Being immunogenetics highly involved in inflammatory and autoimmune disorders, Immunochip includes a dense coverage of the HLA Complex SNPs; this enables deep replication and imputation of the major classical HLA loci (94).

Several autoimmune diseases have already been investigated with Immunochip, such as atopic dermatitis (95), ankylosing spondylitis (96), CD (97), selective IgA Deficiency (98), juvenile idiopathic arthritis (99), MS (100), RA (101), Sj (102), Ps (103), SSc (104); the unique vasculitis analyzed with Immunochip has been Takayasu Arteritis (105). In all these cases, statistically significant immunogenetic variants have been demonstrated; at this time, no Immunochip study has been performed in AAV. Such an analysis may confirm the results obtained from the two GWAS and possibly identifying more associated loci with AAV.

### Targeted sequencing

Targeted sequencing is focused on regions statistically associated by previous studies, such as GWAS. In the case of rare mutations with high penetrance, the addition of more variants and increased sample size may be required, involving deep sequencing in a large number of affected individuals. This approach may add markers to those already known.

Recently has been demonstrated that targeted sequencing of pooled samples increases the chance to efficiently and cost-effectively capture all variation in a more limited target region selectively amplified in multiple DNA samples (106, 107). Such an approach allows efficient use of Next Generation Sequencing technologies, which generate billions of base pairs per experiment, yet introducing challenges in data processing and analysis to discover novel variants and assessing their potential association to disease. In AAV, resequencing will be feasible when several variants will be discovered from different GWAS or Immunochip analysis.

### Whole exome sequencing

In the human genome, there are about 18,000 genes, which contain exons and introns. Exons are short, functionally important sequences of DNA which, together, represent only the portion of the genome that is translated in protein. Whole Exome sequencing (a targeted exome capture) is a recent strategy designed to sequence only the coding regions of the genome (which represents 1% of the human genome or about 30 megabases); this is an effective method alternative to whole genome sequencing, cheaper and less complicated in the bioinformatical/statistical analysis.

The whole exome sequencing potentially may identify the coding variants responsible for both mendelian and common diseases (108).

Whole exome sequencing study may be applied to AAV cases for several topics, such as for familial cases, the investigation of extreme phenotypes, for the finding of mutations or rare variants of already known or novel susceptibility genes and for pharmacogenomic study. Furthermore, the increasing number of susceptibility loci will improve bioinformatic pathway analyses. Investigation of genetically modified animals genetically engineered with identified susceptibility genes may also provide more knowledge to the pathophysiology and potentially validate novel treatment targets.

The elucidation of these novel components may have clinical relevance, as they can be included in genetic-risk modeling approaches. Moreover, they might represent novel biomarkers for AAV, enabling physicians to diagnose patients at risk, preferably before the onset of symptoms, which would greatly reduce the overall cost to society and the burden on patients.

Most importantly, the causal variants, or other molecules that have been identified as playing a role in the same pathway, represent new potential therapeutic targets, not only for AAV but also other autoimmune diseases.

### Meta-analysis and the -omics

Meta-analysis is an important tool to increase the statistical power and analyze the effect of gene variations across groups of different ancestries. A huge amount of GWAS data is becoming available, opening the possibility that future meta-analyses will estimate effect sizes, determining which genes may predispose to different subsets of AAV. Predictive mathematical models integrating the contributing loci may also be helpful. In addition, it is necessary to understand how specific genetic variants are responsible for the association and the biological effect. Fine mapping, targeted sequencing, transcriptomics, proteomics, and metabolomics may be the future goal for being able to stratify risk patients to truly develop a personalized approach to care.

## Conflict of Interest Statement

The authors declare that the research was conducted in the absence of any commercial or financial relationships that could be construed as a potential conflict of interest.
